# Hypergraph Representation Learning with Weighted- and Clustering-Biased Random Walks

**DOI:** 10.3390/e27101072

**Published:** 2025-10-15

**Authors:** Li Liang, Shi-Ming Cai, Shi-Cai Gong

**Affiliations:** School of Sciences, Zhejiang University of Science and Technology, Hangzhou 310023, China; 122121@zust.edu.cn (L.L.); wangwen23560@gmail.com (S.-M.C.)

**Keywords:** hypergraph representation learning, node classification, neural networks, random walk

## Abstract

Hypergraphs are powerful tools for modeling complex systems because they naturally encode higher-order interactions. However, most existing hypergraph representation-learning methods still struggle to capture such high-order structures, particularly in heterogeneous hypergraphs, which results in suboptimal performance on structure-sensitive tasks such as node classification. This paper presents WCRW-MLP, a new framework that integrates a Weighted- and Clustering-Biased Random Walk (WCRW) with a multi-layer perceptron. WCRW extends second-order random walks by introducing node-pair co-occurrence weights and triadic-closure clustering bias, enabling the walk to favor structurally significant and locally cohesive regions of the hypergraph. The resulting walk sequences are processed with Skip-gram to obtain high-quality structural embeddings, which are then concatenated with node attributes and fed into an MLP for classification. Experiments on several real-world hypergraph benchmarks show that WCRW-MLP consistently surpasses state-of-the-art baselines, validating both the efficacy of the proposed biasing strategy and the overall framework. These results demonstrate that explicitly modeling co-occurrence strength and local clustering is crucial for effective hypergraph embedding.

## 1. Introduction

Hypergraphs are generalizations of traditional graphs and provide a powerful framework for capturing complex, higher-order relationships. They are widely applied in diverse domains, including social networks [1], bioinformatics [2], and recommendation systems [3]. In contrast to traditional graphs, which represent pairwise interactions through edges connecting two nodes, hypergraphs can capture many-to-many relationships, thus enabling a more effective modeling of complex and diverse interactions. This unique ability has driven growing interest in hypergraph representation learning, which aims to embed nodes into a latent space while preserving the structural and relational properties of hypergraphs. By leveraging the ability to capture nonlinear and higher-order interactions, hypergraph representation learning offers a powerful approach for analyzing real-world networks.

Recent studies on hypergraph representation learning predominantly employ neural network-based approaches [4], utilizing advanced neural operators, such as convolutional layers, attention mechanisms, and spectral transformations, to learn node embeddings. These advancements have led to the widespread adoption of spectral-based and message-passing architectures, establishing them as foundational methods in hypergraph representation learning. Despite their widespread recognition, these methods present significant limitations. Spectral methods struggle with scalability and are limited by their dependence on global assumptions, while message-passing methods often lose fine-grained structural nuances due to their simplifications of the hypergraph topology. More importantly, both frameworks generally assume uniform or symmetric influence within hyperedges, failing to distinguish subtle variations in node importance or cohesion within hypergraph structures.

An alternative yet underexplored direction is the use of random walk-based methods for hypergraph representation learning. Unlike message-passing architectures, random walks simulate localized paths through the network, offering a dynamic and data-driven mechanism to explore both micro- and macro-scale connectivity. In graph-based contexts, methods such as DeepWalk [5] and Node2vec [6] have demonstrated that random walks, when properly biased, can capture semantic similarities and structural equivalence. Extending this concept to hypergraphs is nontrivial but promising: random walks allow the model to implicitly learn the underlying high-order connectivity patterns without explicitly flattening or transforming the hypergraph into a pairwise form. Furthermore, random walk strategies are inherently flexible—they do not rely on strong assumptions about homophily or hyperedge uniformity. By incorporating node pair co-occurrence weights and clustering coefficients as biases in the transition process, we can more accurately model the structural influence of hyperedges. This opens up the possibility to focus walks toward semantically cohesive regions or diffuse through sparser, weakly connected zones, depending on the task. Thus, we propose a novel framework, WCRW-MLP. In the WCRW part of the framework, we extend the traditional second-order random walk strategy to effectively capture the complex structural features of hypergraphs. The WCRW uses weights of node pairs within hyperedges and biases derived from the triadic closure clustering coefficient [7] of hyperedges. The weights of node pairs represent the co-occurrence frequency of these pairs within all hyperedges, effectively emphasizing the relative importance of different nodes within a given hyperedge. The triadic closure clustering coefficient quantifies the degree of interconnection between hyperedges by measuring how often nodes in one hyperedge participate in other hyperedges to complete closures, thereby capturing latent and complex structural information in the hypergraph. We combine this weight with the clustering coefficient and the traditional second-order random walk method to compute the transition probabilities between nodes. Based on these probabilities, we perform random walks on the hypergraph to generate random walk sequences. Then we input these sequences into the Skip-gram framework to obtain structural node embeddings, effectively capturing and representing the structural properties of the hypergraph. Subsequently, the resulting structural features are combined with the original node attributes and input into a neural network, such as a multi-layer perceptron (MLP). Specifically, the node embeddings generated in this framework seamlessly integrate both structural and attribute features, which can be further utilized for downstream tasks, such as node classification. The proposed method has been extensively validated through experiments, demonstrating its superiority over the state-of-the-art methods across seven benchmark datasets.

The remainder of this paper is organized as follows: Section 2 reviews related works. Section 3 introduces the relevant symbols and definitions related to hypergraphs. Section 4 presents the proposed methodology. Section 5 outlines the experimental setup and discusses the results. Finally, Section 6 concludes the paper.

## 2. Related Works

Hypergraph representation learning aims to embed hypergraphs into a low-dimensional space while preserving their intrinsic structural and relational properties. This section presents an overview of several prominent neural network-based approaches for hypergraph representation learning.

Among neural network-based methods, hypergraph convolutional networks are widely used for hypergraph embedding. Hypergraph convolution is generally divided into two categories: spatial convolution and spectral convolution [8,9]. Spectral convolution leverages the Fourier transform to map graph signals into the spectral domain, facilitating operations such as denoising and smoothing, as demonstrated by HyperGCN [10]. In contrast, spatial convolution aggregates node features directly in the spatial domain using message-passing layers, such as Message Passing Neural Networks (MPNNs) [11]. In this iterative process, nodes gather information from local neighborhoods and progressively incorporate broader structural information. In addition, there are also some spatial convolution methods, such as HGNN [12], UniGCNII [13], and AllSet [14], that utilize a two-stage message-passing process. However, the methods often suffer from significant computational overhead, leading to scalability issues when applied to large datasets.

Random walk-based approaches are traditional yet effective methods for graph representation learning. These methods perform random walks on networks to capture structural contexts and generate node embeddings using natural language processing models like Skip-gram [15]. A key advantage of random walk-based methods is their versatility, as they can be easily applied to most hypergraphs with relatively low computational overhead. The inherent properties of hypergraphs also allow traditional graph-based random walk methods to be adapted to them. However, due to the richer semantics of hypergraphs, traditional graph random walk methods face limitations. Therefore, specialized adjustments are required to fully exploit their potential. To address this, methods such as Node2vec [6], Hyper-SAGNN [16], and Hyper2vec [17] have been proposed. For example, hyper2vec introduces a degree bias strategy based on node2vec, which biases walks on the hypergraph toward nodes with either high or low degrees. However, the degree of a node only reflects its number of neighbors, which may lead to walk sequences that lack sufficient comprehensiveness, thus affecting the quality of hypergraph embeddings. To address this limitation, our method incorporates the triadic closure clustering coefficient as a biasing factor, prioritizing walks on hyperedges with higher or lower clustering tendencies. This strategy generates node sequences enriched with semantic information, which enhances the quality of node embeddings and allows for more effective capture of the hypergraph’s structural information.

## 3. Preliminaries

A hypergraph H=(V,E) consists of a node set $V=\{v_{1},v_{2},\ldots ,v_{n}\}$ and a hyperedge set $E=\{e_{1},e_{2},\ldots ,e_{m}\}$, where each hyperedge is a non-empty subset of nodes.

It is worth noting that the random walk is performed between nodes, and the strength of the association between node pairs within the same hyperedge differs. Therefore, we use the co-occurrence frequency of node pairs within hyperedges to reflect this relationship. The weight $w_{uv}$ for a node pair (u,v) in the hypergraph is computed as follows:

(1) $$w_{uv}=\sum_{e\in E}\delta \left(u,v\in e\right)$$

where

(2) $$\delta \left(u,v\in e\right)=\left\{\begin{array}{cc} 1, & \mathrm{if}\;\{u,v\}\subseteq e\\ 0, & \mathrm{otherwise} \end{array}\right.$$

The triadic clustering coefficient based on hyperedges quantifies the likelihood that two neighbors of a node *u* are also connected through shared hyperedges. It is computed as follows:

(3) C(u)=∑v,w∈N(u)1{∃e∈E:{v,w}⊆e}|N(u)|2

where N(u) represents the number of neighbors of *u*, defined as the nodes that share at least one hyperedge with *u*. The denominator |N(u)|2=|N(u)|·(|N(u)|−1)2 is the total number of possible node pairs in the neighbors of *u*.

This definition captures the tendency of neighbors of *u* to form triadic closures through shared hyperedges, where higher values of C(u) indicate stronger clustering tendencies.

## 4. Methods

**Figure 1:**
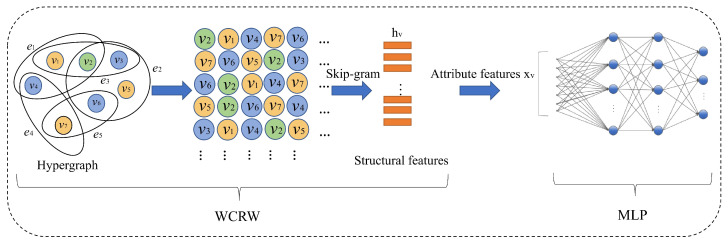
Framework of the proposed WCRW-MLP. We input the hypergraph into the WCRW strategy, which generates random walk sequences to capture structural features. These sequences are processed by the Skip-gram model to produce structural embeddings ($h_{v}$), and then concatenated with attribute features ($x_{v}$). The combined features are input into an MLP for classification, generating the predicted node categories.

Figure 1 gives an overview of the proposed WCRW-MLP framework. The pipeline begins with a WCRW, which extends the conventional second-order walk by injecting two kinds of bias: node-pair co-occurrence weights and clustering coefficient derived from triadic closure. These biases reshape the transition probabilities so that the walker preferentially explores structurally important and locally cohesive regions of the hypergraph. The resulting walk sequences are processed with Skip-gram to obtain structural node embeddings. We then concatenate each structural embedding with the corresponding attribute vector, yielding a unified representation that combines structural and semantic information. Finally, the fused features are fed into a multi-layer perceptron (MLP) for training and inference, producing the predicted node labels.

### 4.1. The Second-Order Random Walk

The key to generating random walk sequences in hypergraphs lies in defining the transition probability from the current node to the next adjacent node. In contrast to the uniform first-order random walk used in DeepWalk [5], the second-order random walk introduces two important parameters: the return parameter *p* and the in-out parameter *q*. These parameters enable the random walker to move to the next node *v*, not only based on the current node *x*, but also considering the previous node *s*. When embedding the hypergraphs, the second-order random walk is more effective in balancing homophily and structural equivalence, thereby improving its ability to capture both local and global structural patterns within the hypergraphs. For a previous step s→t and a candidate next node *v*, the second-order transition probability is calculated as follows:

(4) $$\beta \left(s\to t\to v\right)=\left\{\begin{array}{cc} p^{-1}, & \mathrm{if}\;v=s\;\left(\mathrm{return}\right)\\ 1, & \mathrm{if}\;v\in N(s)\setminus \{t\}\;\left(\mathrm{adjacent}\right)\\ q^{-1}, & \mathrm{otherwise}\;\left(\mathrm{explore}\right) \end{array}\right.$$

here *p* controls the probability of returning to the previous node *s*, while *q* tunes the preference for exploring nodes farther away from *s*. N(s) denotes the neighbor set of the node *s*.

The described strategy relies on the interplay between parameters *p*, *q*, and 1. When $q<\min\left\{p,1\right\}$, the random walker exhibits a higher tendency to explore nodes farther away from the current vertex *t*. This behavior reflects the characteristics of depth-first search (DFS), encouraging the walker to traverse deeper into the network. Conversely, when $p<\min\left\{q,1\right\}$ or $1<\min\left\{p,q\right\}$, the random walker is biased towards revisiting the previous vertex *s* or exploring nodes closer to *s*. Such walks focus on the local neighborhood of the starting vertex, resembling breadth-first search (BFS) behavior by providing a more localized perspective.

### 4.2. Biased Second-Order Random Walk with Weighted and Clustering Coefficients

Building on the second-order random walk strategy described above, adjusting the return parameter *p* and the in-out parameter *q* allows the random walker to favor either depth-first search (DFS) or breadth-first search (BFS). However, certain limitations persist. Both DFS and BFS introduce specific negative biases [18,19], which are especially pronounced in hypergraphs due to the highly skewed node degree distribution. These biases hinder the ability to capture higher-order structural information, while incorporating appropriate positive biases can help identify specific network characteristics, such as densely clustered regions or unique structural relationships. Existing methods, such as the degree-biased random walk model proposed in NHNE [20], select nodes based on their degrees. However, these approaches are limited in capturing the higher-order relationships and clustering tendencies unique to hypergraphs. Specifically, they fail to fully utilize the multi-node associations inherent in hyperedges and struggle to capture dense local structures in high-density regions.

To address the limitations of conventional DFS/BFS biases in capturing the higher-order topology of hypergraphs, we introduce two principled biasing mechanisms in our second-order random walk: node-pair co-occurrence weights and clustering coefficient-based preferences. First, for each node pair (u,v), we compute a co-occurrence weight $w_{uv}$ that counts how many hyperedges contain both nodes. This formulation is supported by Zhou et al. [7], who showed that weighted projections of hypergraphs using node pair co-occurrence frequencies preserve group-level interactions. Recent work by Wen et al. [21] further demonstrates that co-occurrence-based edge weights in hypergraph projections enable accurate downstream learning and reconstruction. Second, we introduce a clustering bias term ψ(v) based on the triadic closure coefficient C(v), which quantifies the tendency of a node’s neighborhood to form triads. Biasing random walks using C(v) has been shown to enhance structural fidelity in node embeddings. For example, Nie et al. [22] demonstrate that incorporating local clustering into walk probabilities improves link prediction accuracy by guiding walkers toward cohesive regions. Similarly, DynamicTriad [23] leverages triadic closure mechanisms to learn dynamic representations faithful to real-world evolution patterns.

**Figure 2:**
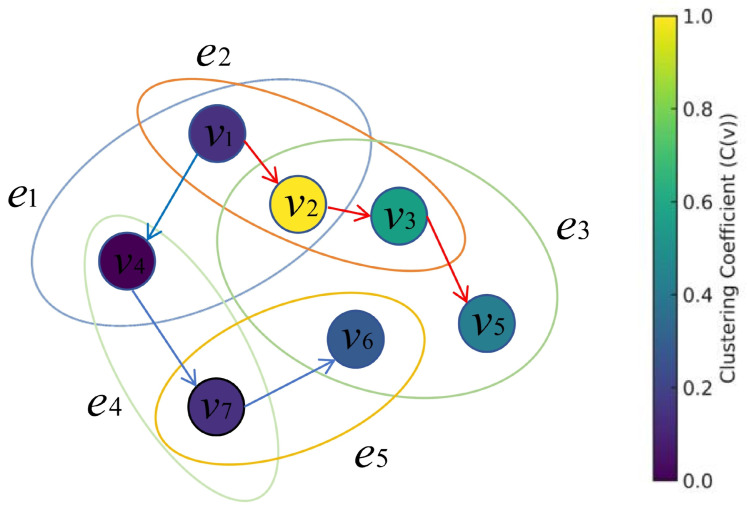
An illustrative example of a hypergraph used in WCRW. Node colors indicate the triadic clustering coefficient.

To bias the random walk towards regions with varying clustering levels, we introduce a bias coefficient ψ(v), which depends on the clustering coefficient of *v* and a parameter α. The function ψ(v) is defined as follows, based on the value of α:

(5) ψ(v)=exp(α·C(v))

where C(v) represents the triadic clustering coefficient of node *v*, and α is the clustering coefficient bias parameter. When α>0, the random walk is biased toward nodes with higher clustering coefficients, promoting transitions within locally cohesive subgraphs. This is illustrated by the red arrow paths in Figure 2, where the walker prefers more connected regions with high clustering. Conversely, when α<0, the walker favors nodes with lower clustering coefficients, facilitating exploration of sparse or loosely connected regions, as shown by the blue arrow paths in the same figure. This flexible mechanism allows for task-specific control over the walk behavior, adapting to the structural characteristics of the hypergraph.

Building upon the previous random walk model, we define the transition probability π as the probability of moving from the current node to a neighboring node, considering both the structural information of the hypergraph and the biases introduced by hyperedge weights and clustering coefficients. This approach allows us to incorporate high-order relationships and clustering tendencies into the random walk process. The transition probability π is adjusted dynamically based on the specific characteristics of the hypergraph, ensuring that the random walk prioritizes nodes that are both structurally significant and relevant to the task at hand. The final definition of the transition probability is as follows:

(6) $$\pi \left(s\to t\to v\right)=\frac{\beta \left(s\to t\to v\right)\cdot w_{tv}\cdot \psi \left(v\right)}{Z}$$

where β(·) represents the second-order random walk bias, which is equal to one when node *t* is the initial node, $w_{tv}$ is the weight between node *t* and node *v*, ψ(v) is the bias coefficient based on the clustering coefficient of node *v* and *Z* is the normalizing factor.

### 4.3. The WCRW Algorithm

Algorithm 1 outlines the complete procedure of the Weighted- and Clustering Coefficient-Biased Random Walk (WCRW) Algorithm. Initially, the hypergraph is preprocessed, and the pairwise node weights, as well as the node clustering coefficients, are computed as specified in Equations (1) and (3). Subsequently, we generate the probability matrix *P* based on the biased second-order random walk model introduced in Section 4.2. Using this matrix, a set of random walk sequences is generated. To optimize the efficiency of walk generation, we employ alias sampling [24] (implementation scripts (Python 3.11.5) are available online https://lips.cs.princeton.edu/the-alias-method-efficient-sampling-with-many-discrete-outcomes/, accessed on 14 March 2025), which enables sampling in constant time O(1). Lastly, the Skip-gram model is utilized to learn the node embeddings.
**Algorithm 1:** The WCRW Algorithm
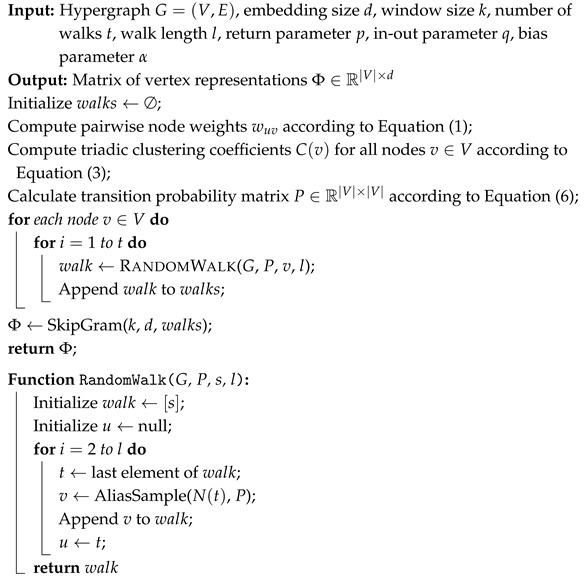


The Skip-gram approach [15], originally designed as a language modeling framework, maximizes the co-occurrence probability of words that appear in close proximity to each other within a sentence. In the present work, this method is adapted to learn node embeddings by predicting the contextual relationships of nodes within the random walk sequences. In our algorithm, the words in a sentence are replaced by nodes within the random walk sequence. Let $f:V\to R^{d}$ denote the center mapping function that maps nodes to embedding vectors, while $f^{\prime }:V\to R^{d}$ represents the context mapping function, and C(t) denotes the context set of node *t*. The optimization problem associated with the Skip-gram model is formulated as follows:

(7) $$\begin{array}{c} \max_{f}\sum_{t\in V}\left(\sum_{c_{i}\in C\left(t\right)}\left(f\left(t\right)\cdot f^{\prime }\left(c_{i}\right)-\log\sum_{u\in V}\exp\left(f\left(t\right)\cdot f^{\prime }\left(u\right)\right)\right)\right) \end{array}$$

Complexity Analysis: The time complexity of calculating the probability matrix *P* is $O(m\cdot k^{2}+m\cdot k^{3})$, where *m* is the number of hyperedges and *k* is the average size of the hyperedges. This is due to the need to compute both the pairwise node weights and the clustering coefficient biases. For the biased second-order random walk procedure, storing the interconnections between neighbors incurs a time complexity of $O(a^{2}\cdot n)$, where *a* is the average number of neighbors, and *n* is the number of nodes. The random walk procedure costs O(t·l·n), where *t* is the number of walks per node and *l* is the walk length. This is due to performing *t* walks per node, each of length *l*, across *n* nodes. Finally, negative sampling allows for the efficient computation of the Skip-gram model.

### 4.4. Feature Fusion and Node Classification with MLP

In this section, we concatenate the structural and attribute features as input to the Multi-Layer Perceptron (MLP) and use it for node classification. First, the structural embeddings of nodes are obtained through the WCRW model to capture high-order structural relationships in the hypergraph. Simultaneously, the original attribute features of nodes are directly extracted from the dataset to preserve their semantic information. To construct a comprehensive representation for each node, we concatenate these two types of features into a unified vector, written as follows:

(8) $$f_{v}=\left[\;h_{v};x_{v}\;\right]$$

where $h_{v}$ and $x_{v}$ denote the normalized structural and attribute features, respectively. This normalization prevents any single feature type from dominating the joint representation after concatenation.

An MLP is designed as the classifier. The input layer of the MLP receives the concatenated feature vectors with a dimension $d_{\mathrm{input}}=d_{\mathrm{struct}}+d_{\mathrm{attr}}$. The hidden layers consist of multiple fully connected layers, where each layer is followed by a ReLU activation function and a dropout layer to improve model expressiveness and mitigate overfitting. The final output layer applies the softmax activation function to produce the probability distribution over node categories. The forward propagation of the MLP can be formulated as follows:

(9) $$z_{v}^{\left(l\right)}=\sigma \left(W^{\left(l\right)}z_{v}^{\left(l-1\right)}+b^{\left(l\right)}\right)\;l=1,2,\ldots ,L,$$

where $z_{v}^{\left(l\right)}$ denotes the output of layer *l*, $z_{v}^{\left(0\right)}=f_{v}$ (input feature vector), $W^{\left(l\right)}$ and $b^{\left(l\right)}$ represent the weight matrix and bias term of layer *l*, respectively, and σ denotes the ReLU activation function.

The training process is trained by minimizing the cross-entropy loss function, which is defined as follows:

(10) $$L=-\frac{1}{N}\sum_{i=1}^{N}\sum_{c=1}^{C}y_{i,c}\log{\hat{y}}_{i,c}$$

where *N* is the total number of nodes, *C* is the number of classes, $y_{i,c}$ is the true label of node *i*, and ${\hat{y}}_{i,c}$ is the predicted probability of node *i* belonging to class *c*.

During training, we use the Adam optimizer to update the model parameters. Additionally, learning rate and weight decay are set to ensure the stability and generalization of the model.

## 5. Experiments

This section evaluates the proposed method via node classification experiments on seven real-world hypergraphs. On two datasets without initial attribute features, we examine the quality of the structural embeddings produced by WCRW. On the remaining five datasets with initial attributes, we further assess the classification performance of the enhanced WCRW-MLP, which builds upon WCRW.

### 5.1. Datasets

We selected seven hypergraph datasets. Among them, two datasets have no initial attribute features: DBLP and IMDb. The remaining five datasets include Cora, Cora-CA, NTU2012, Zoo, and ModelNet40, all of which contain initial attribute features. All of these datasets are publicly available. The dataset statistics are summarized separately in Table 1 and Table 2, where ave(δ(e)) denotes the average hyperedge degree and max(δ(e)) represents the maximum hyperedge degree.

Among the selected datasets, DBLP and IMDb serve as examples of hypergraph benchmarks without initial attribute features. The DBLP dataset captures co-authorship relationships in academic publications, where nodes represent authors and hyperedges correspond to papers, connecting all co-authors. The IMDb dataset represents movie collaborations, where nodes represent actors, and hyperedges connect the top three lead actors of each movie. To ensure reproducibility, we adopted the same preprocessed versions released by NHNE [20], which can be accessed at https://github.com/jeffhj/NHNE/tree/master/graph, accessed on 10 March 2025.

The hypergraph datasets with initial attribute features include Cora, Cora-CA, NTU2012, Zoo, and ModelNet40. The datasets from the citation network Cora and the co-authorship network Cora-CA were obtained from the paper [10]. In the citation networks, all documents cited by the same document are connected via a hyperedge. In the co-authorship network, all documents co-authored by the same group of authors are connected via a hyperedge. In these datasets, the node initial attribute features are represented as the bag-of-words vectors of the corresponding documents, and node labels represent paper categories. Additionally, we utilized two publicly available 3D object datasets from the field of computer vision: NTU2012 [25] and ModelNet40 [26]. NTU2012 consists of 2012 3D shapes from 67 categories, while ModelNet40 comprises 12,311 3D objects from 40 categories. The initial attribute features for these visual objects were extracted using Group-view Convolutional Neural Networks [27] and Multi-view Convolutional Neural Networks (MVCNN) [28]. The construction of hypergraphs follows the methods described in [12,29]. Lastly, for the Zoo dataset from the UCI Categorical Machine Learning Repository [30], the node initial attribute features are combinations of categorical and numerical measurements describing various animals.

### 5.2. Baselines and Settings

Evaluation Groups. Following the dataset grouping in Section 5, we conduct two complementary evaluations: (i) a hypergraph structure-only setting on DBLP and IMDb to isolate the quality of structural embeddings learned by WCRW and (ii) a hypergraph structure + attribute features setting on five benchmark datasets (Cora, Cora-CA, NTU2012, Zoo, ModelNet40) to assess the end-to-end performance of WCRW-MLP that builds upon WCRW embeddings and node initial attribute features. To ensure fair comparisons, we follow the split protocols from the respective prior work [14,20]. For each of the two evaluations, we keep the evaluation metrics consistent within each evaluation setting.

Baseline Selection and Fairness. To ensure fairness and rationality in the comparisons, we clarify the rationale for baseline selection. On the DBLP and IMDb datasets, the experiments are conducted under a structure-only setting without node attributes. Many recent hypergraph representation learning methods rely heavily on attribute information, and applying them in this scenario would require additional assumptions that not only alter the task itself but also result in unfair comparisons. Therefore, we adopt structural embedding methods such as DeepWalk and Node2vec as baselines, since they depend solely on graph topology and their optimization objectives are directly comparable to WCRW. In the structure+attributes setting, however, we conduct systematic comparisons with mainstream and state-of-the-art methods from 2022 to 2025, thereby ensuring both timeliness and comprehensive coverage. This design guarantees fairness with respect to both input modalities and model capacities.

#### 5.2.1. Node Classification in the Structure-Only Hypergraph Setting

We compare the proposed WCRW on the DBLP and IMDb datasets with the following baseline methods: DeepWalk [5], Node2vec [6], LINE [31], Hyper2vec [17], and NHNE [20]. These methods utilize only graph structural information without incorporating node attribute features. We processed the data following the steps outlined in the NHNE [20] method. Some of these methods are traditional pairwise graph embedding methods; thus, we employ clique expansion [32] to convert the hypergraph into a standard graph for these methods. Reasonable parameter tuning was performed for all methods. For random walk-based models such as DeepWalk, Node2vec, Hyper2vec, and WCRW, the window size was set to 5, the walk length to 20, and the number of walks to 10. Unless otherwise stated, the remaining Skip-gram options follow gensim 4.3.2 defaults (negative sampling with negative = 5, initial learning rate alpha = 0.025 linearly decayed to 0.0001, epochs = 5, etc.). For Hyper2Vec, a grid search was conducted over p,q∈{0.25,0.5,1,2,4} and r∈{0,±1,±2,±4,±8,±16}. For WCRW, a grid search was conducted over p,q∈{0.25,0.5,1,2,4} and α∈{−50,−40,…,0,…,40,50}. For LINE, the number of negative samples was set to 5. For NHNE, the number of hidden layer units was set to 32, and the convolution kernel size was set to 3. The embedding dimension was standardized to 32 across all methods. Logistic regression was used as the external classifier, and the model performance was evaluated using micro-F1 and macro-F1 scores with five-fold cross-validation.

#### 5.2.2. Node Classification in the Structure + Attribute Hypergraph Setting

On the five datasets with initial node attribute features, we evaluate WCRW-MLP against several models. These models include HGNN [12], HCHA [33], HNHN [34], HyperGCN [10], UniGCNII [13], UniG-Encoder [35], LE-GCN [29], AllSet (AllDeepSets and AllSetTransformer) [14], ED-HNN [36], FrameHGNN [37], and HyperKAN [38]. To ensure fair comparison, the MLP settings in WCRW-MLP and other methods follow the same training protocol described in [14,36]. Specifically, the data was split into training, validation, and test sets in a 50%/25%/25% ratio. The Adam optimizer [39] with a fixed learning rate and weight decay was used to minimize the cross-entropy loss function. Each model was trained for 500 epochs across all datasets. Dropout was employed to prevent overfitting, and ReLU was selected as the non-linear activation function. Optimal hyperparameters were determined using Optuna [40] with 200 trials. The number of layers was searched within {1,2}, while the hidden dimensions were selected from {64,128,256,512}. The learning rate was tuned from {0.1,0.02,0.01,0.001,0.0001}, weight decay from {0,0.005,0.0005,0.00005}, and dropout rate from {0,0.5,0.7,0.9}. Prediction accuracy was used as the evaluation metric, and for each model, 10 different training–validation splits were conducted to calculate the average accuracy and standard deviation.

### 5.3. Results and Analysis

#### 5.3.1. Node Classification Results of WCRW

As shown in Table 3, we compare the proposed WCRW with five baseline methods to evaluate the quality of hypergraph structural embeddings. On the DBLP and IMDb datasets, WCRW achieves the best results and overall outperforms competing approaches, indicating strong effectiveness for structural representation learning. Compared with conventional random-walk methods, WCRW introduces node–pair weights within hyperedges and a triadic-closure bias based on the clustering coefficient, enabling finer discrimination of association strength and local structural patterns. Furthermore, by emphasizing triadic-closure characteristics in hypergraphs, WCRW effectively captures higher-order relations and alleviates limitations of existing methods in handling hypergraph structure. Benefiting from this design, the model not only adapts well to sparse networks but also accurately models node relations in complex hypergraphs, thereby substantially improving performance on node classification tasks.

#### 5.3.2. Node Classification Results of WCRW-MLP

To further enrich the node embeddings, we proposed the WCRW-MLP framework, which combines the WCRW algorithm with an MLP, fully leveraging both the structural features and attribute features of the hypergraph. As shown in Table 4, we evaluated the performance of WCRW-MLP on five datasets, namely Cora, Cora-CA, NTU2012, Zoo, and ModelNet40, and compared it with some existing methods. The experimental results demonstrate that WCRW-MLP achieves competitive performance on most datasets, showing significant advantages. These findings fully validate its effectiveness and superiority in node classification tasks, further proving the generalization ability and robustness of our method on different datasets.

Building on the above results, we explain why WCRW-MLP achieves superior performance. Conventional hypergraph representations broadly fall into two lines: sampling and embedding approaches and message-passing methods. Both largely model higher-order structure implicitly, which tends to dilute semantic neighborhoods and cause oversmoothing on dense expanded graphs. WCRW addresses this limitation by explicitly injecting two community-salient signals, namely node-pair co-occurrence frequency and triadic closure, into the transition probabilities of a second-order random walk, producing memory-based biased transitions. The second-order walk provides path dependence, the co-occurrence bias strengthens reliable high-frequency co-visits, and the closure bias emphasizes local motif consistency, so that short-context sampling concentrates on structurally coherent and semantically homogeneous clusters. From an objective-function perspective, Skip-gram with negative sampling effectively maximizes the separability between short-context co-visits and random pairs and can be viewed as a low-rank factorization of the windowed co-occurrence matrix. The biased second-order transitions systematically amplify motif-consistent, within-community positives while suppressing incidental co-visits, concentrating the spectrum of the matrix to yield embeddings with stronger linear separability. On this foundation, WCRW-MLP adopts a decoupled fusion approach, concatenating structure embeddings with node attributes and using a shallow MLP to refine the decision boundary. This design avoids the oversmoothing typical of expanded-graph or deep aggregation schemes and, in heterogeneous or sparse regimes, suppresses indiscriminate mixing across weak bridging edges.

In summary, the advantage of WCRW-MLP does not stem from additional model stacking but from the alignment between its second-order memory with explicit co-occurrence and closure biases and the Skip-gram objective, followed by a lightweight attribute-fusion stage. Consequently, gains are more pronounced on structure-dominated datasets, while on attribute-rich datasets the method still achieves stable improvements with better generalization robustness.

#### 5.3.3. Ablation Experiments and Parameter Sensitivity

In this section, we conduct a comprehensive analysis to better understand the effectiveness and robustness of the proposed method. We first perform ablation experiments to isolate the contributions of the weighted-bias and clustering-bias mechanisms in WCRW, then compare the attribute-only MLP with WCRW-MLP, which incorporates both structural embeddings and initial node attributes, in order to highlight the complementary role of structural embeddings. Finally, we investigate the sensitivity of key parameters in the biased random walk process, including walk-related settings and bias coefficients, to gain deeper insights into how different factors influence overall performance.

Ablation Experiments. As shown in Table 5, both single-bias variants—WCRW (Weighted-Bias Only) and WCRW (Clustering-Bias Only)—already achieve competitive performance on DBLP and IMDb, indicating that each bias mechanism is independently effective in enhancing structural representation. However, the complete WCRW model that integrates both bias terms consistently outperforms the single-bias versions, suggesting complementary advantages when simultaneously modeling node co-occurrence strength and triadic closure. These results confirm that the proposed dual-bias design is crucial for fully capturing the structural characteristics of hypergraphs. Table 6 further compares the attribute-only MLP with the proposed WCRW-MLP, which incorporates both structural embeddings and initial node attributes, across five attributed datasets. In the experiments, WCRW-MLP achieves higher classification accuracy than MLP on all benchmarks, with improvements ranging from modest yet consistent gains on NTU2012, Zoo, and ModelNet40 to more pronounced increases on datasets such as Cora. This demonstrates that the structural embeddings learned by WCRW provide complementary information to node attributes, thereby improving overall performance. The comparison underscores that structural signals are not redundant but play a vital role in enhancing the discriminative power of attribute-based models.

**Figure 3:**
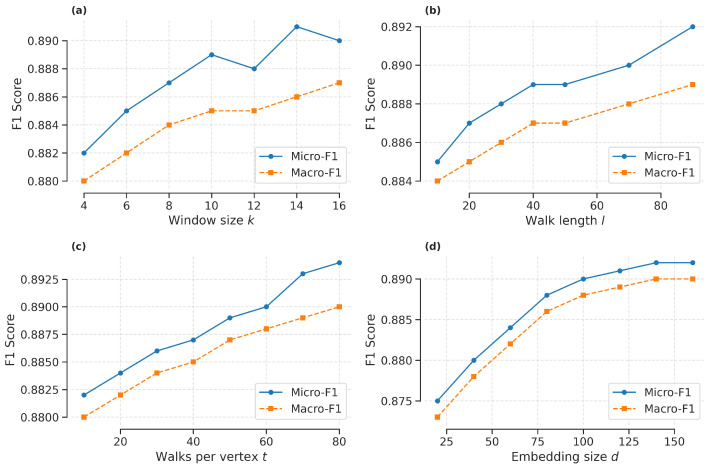
Parameter sensitivity of window size (**a**), walk length (**b**), number of walks per node (**c**), and embedding dimension (**d**) for node classification on the NTU2012 dataset.

**Figure 4:**
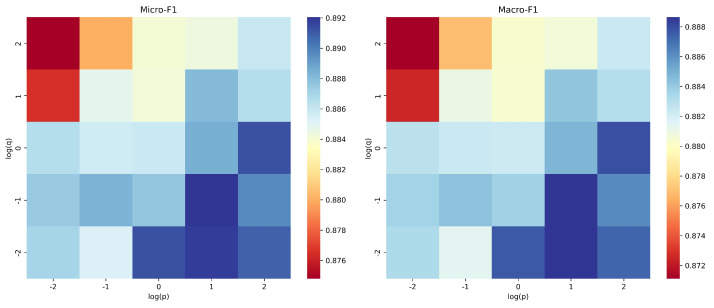
Parameter sensitivity of the return parameter *p* and the in-out parameter *q* for node classification on the NTU2012 dataset.

Parameter Sensitivity. We conducted an empirical evaluation of the parameter sensitivity of WCRW-MLP for node classification on the NTU2012 dataset, focusing on common random-walk parameters: window size *k*, walk length *l*, number of walks per node *t*, embedding dimension *d*, and clustering coefficient bias α. We keep other parameters constant to ensure a fair evaluation. As shown in Figure 3, when the window size *k* increases from 4 to 10, classification performance improves because larger windows capture richer contextual information. However, excessively large window sizes can introduce noise. Similarly, as the walk length *l* increases, performance steadily improves, reaching optimal results at a moderate value. However, excessively long walks may incorporate irrelevant nodes, reducing the model’s effectiveness. Increasing the number of walks per node *t* improves performance, but it plateaus once a certain number of walks is reached. This demonstrates that a sufficient number of walks is critical for capturing structural patterns, but excessively high values add computational cost without meaningful gains. For the embedding dimension *d*, lower-dimensional embeddings limit the model’s representational power, while excessively high dimensions increase the risk of overfitting and computational cost. An embedding dimension of approximately 128 strikes a balance between representation quality and computational efficiency. And then we analyze two important parameters of biased random walks: the return parameter *p* and the in-out parameter *q*. The x-axis denotes the logarithm of *p*, and the y-axis denotes the logarithm of *q* (use 2 as the base of the logarithm), obtained under the optimal parameter setting of the first-order random walk. The heatmap visualizations in Figure 4 show that when p>1 and q<1, the biased random walk achieves optimal performance by balancing local and global structural information.

**Figure 5:**
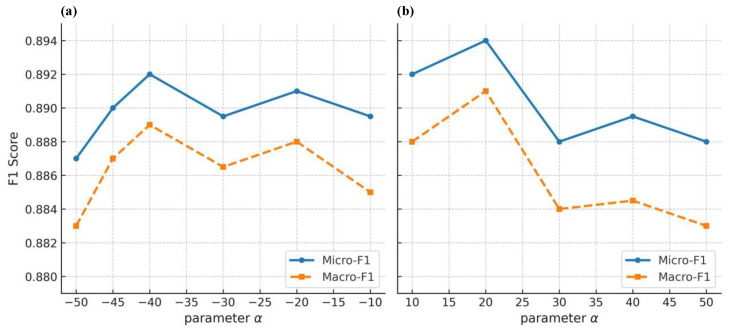
Effect of the clustering coefficient bias α on node classification on the NTU2012 dataset. (**a**) Negative bias (α<0); (**b**) Positive bias (α>0).

Finally, we explore the influence of the clustering coefficient bias α, as shown in Figure 5. The results indicate that the optimal parameter value for node classification lies in the range of α>0. Furthermore, it was observed that the difference in classification results between positive and negative values of α is not significant, which may be attributed to the size of the hypergraph. In cases where the hypergraph has a relatively small number of nodes, longer walk length. and a higher number of walks are typically employed to capture sufficient structural information. Under these conditions, the generated random walk sequences comprehensively encode the hypergraph’s structural information, leading to minimal differences in classification performance between positive and negative values of α. However, when the hypergraph contains a larger number of nodes, the length of the random walk sequences and the repetition counts of nodes are generally constrained to smaller values due to computational complexity considerations. In such cases, the influence of the sign of α on the classification results becomes more pronounced. Further analysis reveals that smaller positive values of α yield better classification performance, whereas larger values of α may interfere with other parameters, thereby affecting the effectiveness of the biased random walk strategy.

Limitations. Our results indicate consistent gains from combining weight-aware and closure-aware biases within a second-order walk, yet several factors delimit the scope of these findings. Relying on the clique expansion collapses higher-order relations into pairwise links and may bias the representation toward nodes involved in large hyperedges. The bias terms are hand-crafted and governed by fixed hyperparameters, which favors interpretability but may be suboptimal across domains. On ultra-large, dense hypergraphs, computing the triadic closure metrics and maintaining sampling structures for second-order random walks incur substantial time and memory costs, necessitating trade-offs among accuracy, speed, and memory footprint.

## 6. Conclusions

In this paper, we propose a novel framework, WCRW-MLP, which combines a biased second-order random walk strategy (WCRW) with a MultiLayer Perceptron (MLP) for enhanced hypergraph representation learning. The random-walk component incorporates pairwise node weights and triadic-closure coefficients to capture higher-order structural relationships and local cohesive patterns in hypernetworks, while the overall framework integrates hypergraph structural embeddings with node attribute features for downstream node classification. Extensive experiments on seven real-world hypernetwork datasets demonstrate the effectiveness of the proposed strategy and framework. Our method outperforms other baselines in classification tasks, highlighting its advantages and adaptability in hypergraph representation learning. WCRW-MLP can be applied to real-world systems with multi-entity interactions, such as recommendation, collaboration, and multi-omics networks, where co-occurrence and local closure patterns naturally emerge. These structural regularities enable the framework to effectively capture group-level associations and behavioral patterns. Although computing local closure introduces additional cost, the overall complexity remains manageable because the model mainly relies on pairwise node–hyperedge relations rather than dense graph expansions. In practice, sampled or approximate estimation of closure, together with parallel walk generation and other engineering optimizations, can substantially reduce runtime, making the method practically feasible for large-scale hypergraphs. For future work, we will develop native higher-order walking that samples hyperedges and motifs without pairwise compression. In addition, we plan to make the bias terms learnable and data-adaptive and extend the framework to dynamic and inductive settings with time-aware transitions and lightweight encoders. The model can also be expanded to integrate heterogeneous and multimodal attribute features at both node and hyperedge levels, enabling stronger fusion beyond the current MLP design.

## Figures and Tables

**Table 1:** Dataset statistics for benchmarks without initial attribute features.

Dataset	|V|	|E|	Classes	ave(δ(E))	max(δ(E))
DBLP	7995	18,364	3	3.02	29
IMDb	4423	4334	20	2.89	3

**Table 2:** Dataset statistics for benchmarks with initial attribute features.

Dataset	|V|	|E|	Features	Classes	ave(δ(E))	max(δ(E))
Cora	2708	1579	1433	7	3.03	5
Cora-CA	2708	1072	1433	7	4.28	43
NTU2012	2012	2012	100	67	5	5
ModelNet40	12,311	12,311	100	40	5	5
Zoo	101	42	16	7	39.93	93

**Table 3:** WCRW’s results of node classification as micro-F1 and macro-F1 scores (%) on DBLP and IMDb.

Embedding Algorithm	DBLP		IMDb	
	*Micro-F1 *	*Macro-F1 *	*Micro-F1 *	*Macro-F1 *
DeepWalk	87.46	87.03	44.23	15.25
Node2vec	87.66	87.21	44.41	15.42
LINE	81.43	80.80	44.03	12.44
Hyper2vec	88.66	88.25	44.82	15.83
NHNE	89.39	88.93	45.35	17.50
**WCRW**	**90.56** * ± 0.0011	**90.29** * ± 0.0012	**46.19** * ± 0.0024	**17.92** * ± 0.0033

* indicates that WCRW significantly outperforms the best baseline at the 0.05 levels (paired *t*-test).

**Table 4:** WCRW-MLP’s results of node classification as mean accuracy (%) ± standard deviation for each method. For each dataset, the best-performing score is in bold and the second-best is underlined.

Hypergraph Algorithm	Cora	Cora-CA	NTU2012	Zoo	ModelNet40
HGNN	79.39 ± 1.36	82.64 ± 1.65	87.72 ± 1.35	95.50 ± 4.58	95.44 ± 0.33
HCHA	79.14 ± 1.02	82.55 ± 0.97	87.48 ± 1.87	93.65 ± 6.15	94.48 ± 0.28
HNHN	76.36 ± 1.92	77.19 ± 1.49	89.11 ± 1.44	93.59 ± 5.88	97.84 ± 0.25
HyperGCN	78.45 ± 1.26	79.48 ± 2.08	56.36 ± 4.86	85.38 ± 6.23	75.89 ± 5.26
UniGCNII	78.81 ± 1.05	83.60 ± 1.14	89.30 ± 1.33	93.65 ± 4.37	98.87 ± 0.23
UniG-Encoder	81.43 ± 1.37	85.58 ± 1.13	90.42 ± 1.49	98.46 ± 3.71	98.41 ± 0.17
LE-GCN	77.34 ± 1.10	76.60 ± 1.63	89.16 ± 1.13	95.00 ± 4.81	96.68 ± 0.16
AllDeepSets	76.88 ± 1.80	81.97 ± 1.50	88.09 ± 1.52	95.39 ± 4.77	96.98 ± 0.26
HyperKAN	–	–	90.58 ± 1.48	–	98.52 ± 0.18
AllSetTransformer	78.58 ± 1.47	83.63 ± 1.47	88.69 ± 1.24	97.50 ± 3.59	98.20 ± 0.20
ED-HNN	80.31 ± 1.35	83.97 ± 1.55	88.07 ± 1.28	95.77 ± 3.37	98.35 ± 0.20
FrameHGNN	81.51 ± 0.99	85.18 ± 0.69	89.98 ± 2.02	–	98.41 ± 0.18
**WCRW-MLP**	**84.12 ± 1.25**	**86.20 ± 1.10**	**91.25 ± 1.43**	**98.93 ± 3.85**	**98.90 ± 0.23**

**Table 5:** Ablation of WCRW bias mechanisms on DBLP and IMDb for node classification.

Variant	DBLP		IMDb	
	*Micro-F1 *	*Macro-F1 *	*Micro-F1 *	*Macro-F1 *
WCRW (Weighted-Bias Only)	88.46 ± 0.0013	88.27 ± 0.0013	44.93 ± 0.0018	16.18 ± 0.0043
WCRW (Clustering-Bias Only)	89.23 ± 0.0017	89.01 ± 0.0017	45.13 ± 0.0016	16.31 ± 0.0056
WCRW (Both Biases)	90.56 ± 0.0011	90.29 ± 0.0012	46.19 ± 0.0024	17.92 ± 0.0033

**Table 6:** Node classification accuracy (%) of attribute-only MLP versus structure + attribute WCRW-MLP on five attributed datasets.

Dataset	MLP	WCRW-MLP
Cora	77.49 ± 1.43	84.12 ± 1.25
Cora-CA	77.40 ± 1.38	86.20 ± 1.10
NTU2012	89.08 ± 1.58	91.25 ± 1.43
Zoo	94.62 ± 4.51	98.93 ± 3.85
ModelNet40	96.70 ± 0.23	98.90 ± 0.23

## Data Availability

The original contributions presented in this study are included in the article. Further inquiries can be directed to the corresponding author.
